# Mapping infectious disease hospital surge threats to lessons learnt in Singapore: a systems analysis and development of a framework to inform how to DECIDE on planning and response strategies

**DOI:** 10.1186/s12913-017-2552-1

**Published:** 2017-09-04

**Authors:** Shweta R. Singh, Richard Coker, Hubertus J-M Vrijhoef, Yee Sin Leo, Angela Chow, Poh Lian Lim, Qinghui Tan, Mark I-Cheng Chen, Zoe Jane-Lara Hildon

**Affiliations:** 1 Saw Swee Hock School of Public Health, National University of Singapore, Tahir Foundation Building, Block MD1, 12 Science Drive 2, Singapore, 117549 Singapore; 2 Mahidol University, 9th Floor, Satharanaukwisit Bldg, 420/1 Rajwithi Rd, Bangkok, 10400 Thailand; 3 Faculty of Public Health and Policy, London School of Hygiene and Tropical Medicine, Keppel street, London, WC1E 7HT UK; 4 Department of Patient and Care, Maastricht University Medical, PO Box 5800, 6202 AZ Maastricht, the Netherlands; 5 Department of Family Medicine, Vrije Universiteit Brussel, Brussel, Belgium; 6 Institute of Infectious Diseases & Epidemiology, Communicable Disease Centre, Tan Tock Seng Hospital, Communicable Disease Centre, Moulmein Road, Singapore, 308433 Singapore; 7 Biological Resource Centre, Agency for Science, Technology and Research (A*Star), 1 Fusionopolis Way, #20-10 Connexis North Tower, Singapore, 138632 Singapore; 8 John Hopkins University, Center for Communications Programs, 111 Market Place, Suite 310, Baltimore, 21202 MD USA

**Keywords:** Infectious disease outbreaks, Surge capacity and capability, Health planning and implementation

## Abstract

**Background:**

Hospital usage and service demand during an Infectious Disease (ID) outbreak can tax the health system in different ways. Herein we conceptualize hospital surge elements, and lessons learnt from such events, to help build appropriately matched responses to future ID surge threats.

**Methods:**

We used the Interpretive Descriptive qualitative approach. Interviews (*n* = 35) were conducted with governance and public health specialists; hospital based staff; and General Practitioners. Key policy literature in tandem with the interview data were used to iteratively generate a Hospital ID Surge framework. We anchored our narrative account within this framework, which is used to structure our analysis.

**Results:**

A spectrum of surge threats from combinations of capacity (for crowding) and capability (for treatment complexity) demands were identified. Starting with the *Pyramid* scenario, or an influx of high screening rates flooding Emergency Departments, alongside fewer and manageable admissions; the *Reverse-Pyramid* occurs when few cases are screened and admitted but those that are, are complex; during a ‘*Black’* scenario, the system is overburdened by both crowding and complexity. The Singapore hospital system is highly adapted to crowding, functioning remarkably well at constant near-full capacity in *Peacetime* and resilient to *Endemic* surges. We catalogue 26 strategies from lessons learnt relating to staffing, space, supplies and systems, crystalizing institutional memory. The DECIDE model advocates linking these strategies to types of surge threats and offers a step-by-step guide for coordinating outbreak planning and response.

**Conclusions:**

Lack of a shared definition and decision making of surge threats had rendered the procedures somewhat duplicative. This burden was paradoxically exacerbated by a health system that highly prizes planning and forward thinking, but worked largely in silo until an ID crisis hit. Many such lessons can be put into play to further strengthen our current hospital governance and adapted to more diverse settings.

## Background

Increase in trade and travel, as well as ecological and climate changes have made pockets of the world more vulnerable to health system surges caused by Infectious Disease [1] (ID). Singapore is a financial hub and major tourist, and transiting port of South-East Asia [2]. This coupled with an ageing population, and space crunch in Singapore’s major hospitals [3] makes ID Surge planning a central policy issue.

Since the aftermath of SARS (2003), the first major pandemic of 21st century, it is anticipated that many countries still do not have adequate advance emergency planning to deal with outbreaks, including threats of H5N1, H1N1 influenza [4–8]. Some key challenges include shortage of Protective Personal Equipment (PPE), communication, and systems incident management [4, 5, 8]. Systems bottlenecks are the largest crosscutting issue for surge planning and response. For example, according to a study of 29 disasters prior to 9/11 in USA, in only 6% of cases was there a supply shortage and only 2% suffered manpower shortages [9]. The issue therefore is often about getting the right resources to the right places at the right time.

We have undertaken a qualitative study using the Interpretive Description (IntD) approach [10–12], based on in-depth interviews with healthcare (e.g. clinicians, public health specialists) and policy (Ministry of Health) professionals in Singapore. This method seeks to narrate health policy and service improvements through broad-brush qualitative analyses, which relate experiences to an interpreted framework, constructed from what is known to be significant to the issue of interest. Given that surge events can be meaningfully conceptualized [8, 13–15] and are also bound to realities and shifting contexts, the IntD theoretical underpinnings are well aligned to our work. These underpinnings also allowed for the co-construction of meaning between researchers and participants.

Therefore, we aim to produce an evidenced-based set of tools to improve incident management systems informed by narrating accounts of ID outbreaks in Singapore from 1999 onwards (since the Nipah outbreak) [16]. Our objectives are to:

1. Identify the basic conceptual elements of hospital ID surges, combining constructs from the literature [13–15, 17] with those emergent in our data, and situating analysis by describing the backdrop of ID surge events featuring in our context;
2. Describe a typology of ID hospital surge threat scenarios primarily in relation to virulent air/droplet borne diseases - contrasting with vector borne scenarios - and link this to levels of alert for hospital planning and response;
3. Collate lessons learnt from past air/droplet borne ID outbreaks and catalogue these into incident management strategies related to: staffing, space, supplies (or ‘stuff’ sic) [15] and systems [14, 15].

Basic elements of hospital surge events were extracted from literature captured in a systematic review on this topic [14, 15]. These analyses were further verified against key policy literature identified by scoping the World Health Organisation (WHO) and Singapore Ministry of Health (MoH) websites.

## Methods

We used a multiple stakeholder qualitative design (*n* = 35), conducting interviews with largely tertiary and policy sector health care professionals, as well as primary care physicians, Table 1. We report study methods following the Consolidated Criteria for Reporting Qualitative research (COREQ) checklist [18]. Data were collected by a senior social scientist and experienced qualitative researcher (ZH), and trained female researchers (SS, JJ and QT), from clinical, bio-sciences and public health backgrounds. ZH initially lead the interviews, supported by the clinical team members who later co-led them together or conducted them independently.

**Table 1:** Sample characteristics by professional cadre, *n* = 35

	Main functions	N^O^	Years’ experience (mean)	Standard Deviation from the mean
Hospital based staff	Clinicians (C)	8	16	8.59
	Microbiologist (M)	3	17	8.49
	Nurses (N)	7	11.6	9.38
	Operations manager (OM)	3	30.33	3.30
Governance and public health specialists	Board members (BM)	4	26.25	5.40
	Public health practitioners (PHP)	5	15.4	9.41
Primary health care physicians	General Practitioner (GPs)	5	32.2	11.50

The interviews were semi-structured with a narrative component at the start, lasting up to an hour. The narrative component asked interviewees to recount their most memorable surge outbreak, these were hooked onto a historical timeline of known outbreaks (1999, Nipah virus; 2003, SARS; 2005, 2007 and 2013, Dengue; 2008, Chikungunya; 2009, H1N1-Swine flu; 2014 Ebola preparations; 2015 MERS CoV preparations). These included both air and droplet borne, and vector borne diseases.

Related topics structuring the reminder of the interview included: current state of resources; historical opportunities for improvements; decision-making processes. The topic guide was piloted and revisions incorporated, see Supplementary file for a copy of this tool. Given we followed the Interpretive Descriptive theoretical approach, we prompted narrative with very open questions to start but also helped to co-construct the focus of the discussion, anchoring it towards the elements of hospital surging known to be of interest.

Sampling was purposive, seeking maximum variation of healthcare professionals with significant experience managing ID surges; emphasis was on recruiting reputed specialists in the ID field to start with. Most re-structured hospital representatives gave permission for their staff to be approached; Singapore General Hospital (SGH) refused to participate. This included clinical staff working in the Infectious disease, Emergency Medicine and Microbiology department from regional institutions (Changi General Hospital, CGH; Khoo Teck Puat Hospital, KTPH; National University Hospital, NUH; Tan Tock Seng Hospital, TTSH; Ministry of Health, MoH). Participants comprised Public Health Specialists (*n* = 5), Board Members (*n* = 4), Clinicians (*n* = 8), Nurses (*n* = 7), Operations Managers (*n* = 3), and General Practitioners (GPs; *n* = 5). TTSH is the major ID hospital in Singapore, thus 53% of interviews were carried out with participants based at this hospital.

With the help of our collaborators we reached out to a core group of ID specialists in each of the restructured hospitals that agreed to take part in the study by email. Respondents were invited if they had experience working in Singapore health system from 2003 onwards and had ID expertise. Once we had the core group of ID specialists we asked them for recommendations at the end of their interview, or snowballed [19] until the range of participants were recruited, mostly via email invitation. Snowballing was used to gain an entry point for hard-to-reach, very busy professionals, who could not be emailed as a ‘cold call’. Frontline healthcare workers were approached directly at the shift end, with the introduction of senior clinicians or head nurses. General practitioners (GPs) were recruited on ASEAN Dengue day conference held in Singapore.

We stopped participant recruitment once saturation from referrals was reached; 81% of invited interviewees agreed to take part. Interviewees had been in the Singapore healthcare system an average of 20 years. Interviews were conducted in institutional settings; preferably in more neutral meeting rooms, or otherwise in the participants’ offices. The interviews started with defining ID health services surges, which was very broadly stated as a ‘sudden, anticipated or unanticipated escalation in health service usage caused by an ID outbreak’. We also asked participants to define ‘capacity’, and ‘capability’ in the hospital setting. The fuller discussion was then triggered by asking about the most memorable surge experience, and hooked onto a timeline of historic outbreaks.

The majority of the participants met the researchers for the first time during the interview. All participants consented to be audio-recorded for the interview on condition of anonymity. Primary and frontline health-care workers’ interviews tended to be shorter, lasting for 20–35 min for health care workers, whereas senior level participant’s interviews were longer. Rapport was easily established, although we did ask for permission to follow-up with repeat interviews, this was not judged necessary because of the richness of the data and openness of interviewees during the first encounter; member check of analyses and selected quotes was undertaken prior to publication.

Prior to narrative analysis we familiarised ourselves with data by reading and rereading the transcripts, which were then sorted under a Hospital ID surge Framework, which derives its known elements from the key literature [13–17], as well as additional conceptual categories emergent from qualitative coding and sorting of data. JJ and SS organised the data, ZH and SS discussed codes and confirmation of the frameworks components. This included iterative comparisons against our interview data, known elements, repositioning and finally confirming analyses. SS then coded the transcripts according to our objectives for: 1) elaborations of surge events; 2) the typology of surge threats; and 3) the emergent lessons learnt. ZH refined and agreed these analyses.

**Fig. 1:**
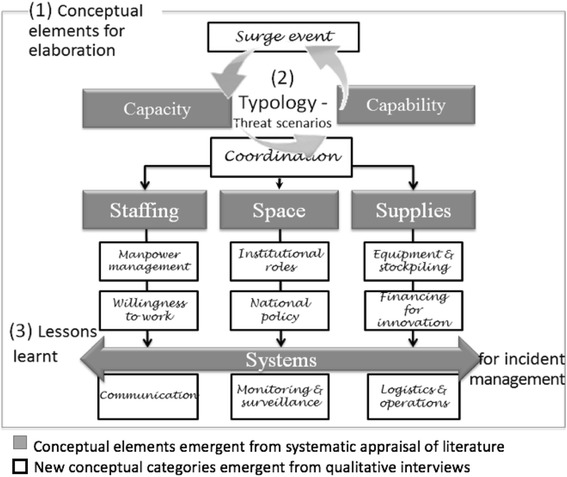
Framework for ID hospital surge planning & response

We report results according to the IntD method using a narrative reporting format [12]. This does not result in a list of themes, but instead in accounts that respond directly to the study objectives. We structure analysis around the conceptual elements presented in Fig. 1 and narrate each in turn, anchored in our data.

## Results

### Objective 1: Identifying the conceptual elements of ID hospital surges

The Hospital ID Surge Framework (Fig. 1) has two core, opposing yet complimentary elements: Capacity and Capability. As one nurse summarised these: *‘So surge [capacity] is a sudden increase in numbers in a short period of time… [and capability] something like in your ability to control [surge events] within your own limits.*’ - N7.

*Capacity* is broadly referred to in respect to crowding, and as such is known as the quantitative arm of surge management [13] and having enough of what is needed such as beds, medicine and people to manage it [14, 15]. Capacity was readily understood by our participants as such, for example: ‘*So I suppose Surge is a statistical term laah, so it refers to incident within a period of time.’ - C2*.

*Capability* is broadly conceptualised by the complexity of disease management. It is the qualitative sister to capacity. It refers instead to the specialised ability needed to understand a pathogen and offer related specialized care [15, 17]. Capability was anchored in competencies and necessarily the ‘knowability’ of a pathogen, and quality and safety procedures. These were notably based on institutional memory: *‘So when you build these capabilities and you have an institutional memory, then you are better able to react and respond more intuitively to the next sets of uncertainties. So, I think that is what we have in place.’ - BM3.*

The Framework for ID hospital surge planning & response, Fig. 1, anchors these two known core concepts as well as the known structure of *staff, space, supplies* and *systems* [14, 15]: the ‘four Ss’ (boxed in grey), to our narrative analysis. We then present new concepts that emerged beyond, across, and within these existing known elements.

The Framework shows the concepts that emerged inductively from the interviews (boxed in white). We found the concept and understanding of a Hospital *Surge Event* itself important. We therefore begin the narration of the framework by elaborating the nature of Surge Events in our setting, as an extension of our first Objective and to situate our findings. These events are ultimately related to *Typologies*, described by Objective 2, which are represented by more or less emphasis on capacity and capability; all of which lead squarely to *Coordination* with respect to the ‘four Ss’. The bulk of our new concepts largely include sub-components of the ‘four Ss’, which will be further elaborated under the lessons learnt Objective 3.

#### Situating the analysis: Hospital surge events in Singapore

The first outbreak on our timeline was the *Nipah* virus in 1999. However, for this outbreak it emerged that its impact wasn’t felt much due to the small number of cases, non-complexity of treatment, and early containment [16]. The most memorable surge event experienced by our participants was almost always *SARS* (2003). SARS was the first ID outbreak that caught people completely off guard, and became crystalized as something that changed ID practices thereafter. The slow-to-be-understood and complexity of the disease was its most painful feature: *‘In the beginning SARS was difficult. So… it evolved and then, you know, such that…it’s so meticulous until you have to have goggles and eyewear and visors’ - PHP5.*

Though SARS was unanimously named and discussed as the most dramatic surge event, it was also, somewhat paradoxically, characterised by an unusually ‘empty hospital’. SARS served as a turning point in the ID surge preparedness in Singapore and was said to have taught many lessons, used to curtail other epidemics. For example, one Clinician noted: *‘Essentially SARS shook us in how we practice, the whole hospital, right from emergency department all the way to the wards and the ICU. So, we actually have a set of protocols, on how things are to be done…so the concept was fresh from SARS’ - C1.*

During the *H1N1* (2009) outbreak an overwhelming number of patients were described as crowding the hospital Emergency Department (ED) during the earlier containment phase. Because H1N1 wasn’t as complex a disease as SARS, hardly any patient was admitted in ICU. As one Nurse described it: *‘The difference in H1N1 was […] because. I remember that the peak period of H1N1 we were screening about 600 cases every day. Yah, so the surge was the challenge then… It was the turnaround time for the screening results to be out. So that took about between 6 to 12 hours. And imagine having a few hundred people in front of you for 6 to 12 hours and they getting fidgety and some of them were called from airport and brought here, yah’ - N1.*

Despite this extreme patient load, the H1N1 surge was described in far less dramatic terms compared to SARS. Some of the clinicians mentioned that the containment policy for H1N1 initially was an overreaction and other nations had a better response to surge by not initiating mass screening policies. Correspondingly, *Dengue* (2005, 2007 and 2013) outbreaks were described as having the lowest impact. Some GPs reported extra loads, but generally hospital staff said they system was able to cope well, since this flow of patients tended to be expected and not much over the norm, for instance: ‘*Actually, I didn't feel that much, you know, day in day out we just work. Of course, there are a number of dengue patients coming in… I don't feel stress because of the dengue patients’- N2.*

The *Chikungunya* outbreak (2008) was described in comparable terms, also a vector borne disease, for which the transmission tended to be slower due to prevention at source. These IDs were related by our respondents to seasonal flu: which is easily transmittable in theory yet in practice does not cause volumes of cases because of early preventative measures, vaccines and self-containment. Plus, these IDs were described as easily managed once diagnosed: *‘Dengue, you know, and because of during the flu season when we have more cases of pneumonia, respiratory diseases coming in. So that I think we experience it regularly.’* - OM3. We observed a system highly adapted to capacity threats.

### Objective 2: Describing a typology of ID hospital surge threat scenarios to link to alert levels for planning and response

**Fig. 2:**
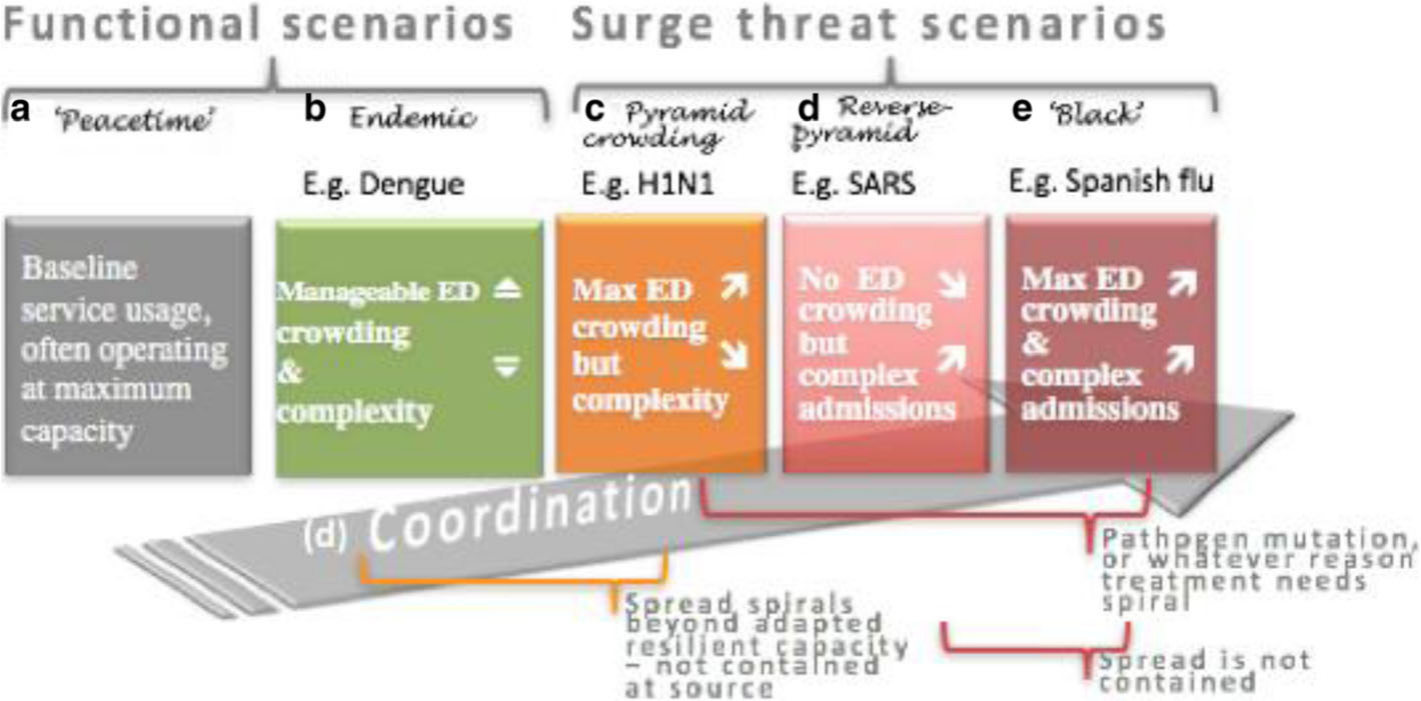
Typology of hospital surge threats scenarios

The types of functional vs. threat ID surge scenarios in our context are summarised in Fig. 2. In our typology of threat scenarios, it was the combination and extent to which capacity and capability were experienced that determined the surge threat intensity.

#### Peacetime scenario

Our participants talked about ‘Peacetime scenario’ (Fig. 2a) which was described as a time where no ID outbreak was occurring. Nevertheless, this the Singapore setting this was experienced as a system that was already stretched for capacity: *‘Hospital bed occupancy rates are usually in the red zone, somewhere around 85-95% on a daily basis, so very little capacity’*- *C7*.

#### Endemic scenario

It was interesting that Endemic or seasonal scenarios (Fig. 2b) like Dengue or flu were consistently, and by all hospital based cadres, differentiated from what was considered threatening surges: ‘*Everyday fluctuations, or endemic diseases, we don’t actually consider it as a surge’ – C3.* Therefore, the Singapore system has become highly resilient in terms of adapting to lack of space and crowding. As long as hospital demand spikes were related to well-known IDs and prevented from exploding at source, they were viewed as manageable.

#### Pyramid crowding scenario

Threats were experienced when the ID need for screening spread beyond adapted, resilient capacity. The H1N1 outbreak was described thus. This outbreak takes the shape of a Pyramid crowding scenario (Fig. 2c), shrinking upward from a high screening volume to fewer and fewer admissions: *‘The number of patients coming to the hospital forms this space, alright [holds hands wide apart]. The number of patients that really are at risk is this big [shows a smaller space]. And then it goes up, it goes up, and finally there at the top are the patients that are infected [shows very small space]’ – BM4.* The size of the Pyramid tiers was seen to also inform the decision to refer for screening in the first place, and contact tracing policies, which can themselves be either overblown or not stringent enough.

#### Reverse-pyramid scenario

Most mentions of capability came from narratives on SARS, which can be described as a (Fig. 2d) Reverse-Pyramid scenario. Here the issue was not ED crowding but the complexity of managing the disease that was the challenge, as one participant remarked: ‘*It is not business as usual. [although] In fact, during SARS, the hospital is actually quite empty*’ - BM4. Despite little crowding it was this service surge demand that caused the most fear, disruption, casualties and confusion in our setting.

#### Black scenario

Prophesized ‘Black’ scenarios (Fig. 2e) consisted of crowding and high caseloads, combined with little ability to manage their complexity, as described here: *‘What happens if you exceed that [past predicted capacity and capability], and I think that is Spanish Flu revisited type of scenario, which is classically a Black scenario, which everybody knows if it happens, you know, life ends as it extends.’ - BM3.* Spanish flu (1918) [20] was not contained at source, nor easily treatable, so exploded into an international pandemic.

#### Coordination

Hence coordination (Fig. 2d) emerged as a key concept, based on the real fears that a lack of both capacity and capability could lead to Black outcomes. Coordination will vary according to which threat scenario is being experienced. Specifically, the threat level as determined by the combination of crowding and complexity.

In the case of SARS quickly actioned contact tracing curtailed transmission at source, as did a stringent containment response and hospital infection control, this prevented the Black scenario in which both the front end and back end of hospital services are overloaded with complex cases. H1N1 could also have exploded into a blacker scenario had the pathogen mutated or the disease somehow been harder to treat. In our settings Dengue is very well managed at source but in other less prepared setting outbreaks may escalate to pyramids with high screening rates and also wider middle tiers, making more admissions as the top tier.

Despite addressing very real fears, ID surge planning was described as immensely burdensome: *‘The fear of Ebola is a much bigger wave compared to the disease itself.’ - BM1*. Much emphasis was placed on managing cases *before* they get out of control - if capability is effective, capacity vulnerabilities can be reduced. Hence, a lot of resources and efforts were very visibly exhausted with this aim in mind, put simply: *‘You know then I would say, you don’t need to do anymore here. There is nowhere better prepared than here.’ - C5.*

Therefore, being on alert was highly prized, yet little coordination appeared to go into sharing this burden between hospitals, or clearly defining threats and related needs and priorities within coordination practices. Explicit points of reference to inform coordination also appeared to be lacking. Participants from hospitals follow detailed protocols such as – ‘*Ebola was something entirely different. So that’s why they have to come up with err new protocols’ - N6 –* which requires a lot of effort to be tailored to each looming ID surge event.

Yet there is no alert system, providing definitions of surge threats for hospitals, their staff and governors. Another senior participant described this gap thus: *‘if I fast forward that to the current Ebola, now we have to basically deal, number one [first and foremost], with a very considered view of* ***what exactly is a threat*** *[emphasis our own]; so, when I was overseeing the initial Ebola requirements for the hospitals, there were a lot of barriers that were primarily driven by conventional [and implicitly inaccurate] definitions [of threats].’ - BM3.*

The current alert system used in Singapore is called: ‘Disease Outbreak Response System Condition’ ‘DORSCON’ [21], which is initiated to help guide public containment, contact and to some extent screening behaviours, based on the perceived danger of spread from the ID, within and beyond Singapore. This system is based on a community and boarder control perspective, and is a useful in accounting for transmission modes beyond hospital settings.

### Objective 3

Mapping lessons learnt to surge threats to improve coordination.

The lessons learnt are anchored onto the recognised incident management domains. In this set of analyses, we elaborate the sub-categories for each of these areas, as listed in Fig. 1:

- Staffing: Manpower management and willingness to work;
- Space: Institutional roles and national level policy;
- Supplies: Equipment and stockpiling;
- Systems: Financing for innovation, communication, monitoring and surveillance, as well as logistics and operations.

**Fig. 3:**
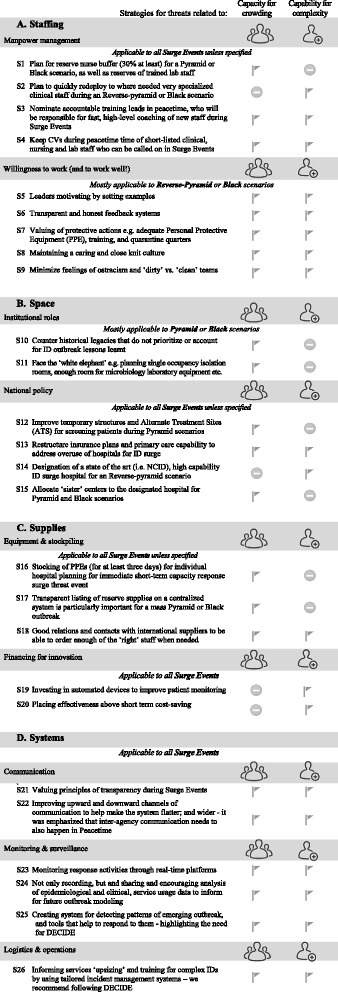
Mapping lessons learnt to surge threat scenarios

In Fig. 3 we catalogue corresponding strategies from lessons learnt relating to each of these categories and sub-categories. Strategies are indicated as S1 to S26; we narrate them below according to the IntD approach in relation to our threat scenarios and capacity vs. capability challenges.

#### Staffing

*Staffing* is a challenge during any surge threat, since both sufficient and adequately trained staffs need to be mobilised. After SARS, ID physicians in Singapore tripled in numbers [15] but still it was feared that in the event of another potential Black scenario there may be shortfall. *Manpower management* during an outbreak requires an increase in the staff/patient ratio. The first strategy noted by our participants was (S1) planning for an increase in nursing staff in particular – a reserve buffer of 30% was considered ideal by operations managers, to relieve pressure during Pyramid or Black threat scenarios: ‘*So because I think it depends on what type of outbreak and therefore what kind of deployment you need la basically. But all I can say is that so far, besides the SARS […] we are not needed any additional medical manpower. It is only the nursing manpower… So, we just have to supplement whatever we need if there is any infectious disease outbreak basically’ - OM3.*

On the other hand, (S2) specialist clinical staff were see as needing redeploying the advent of a Reverse-Pyramid or Black scenario. Senior participants mentioned that during ID outbreaks they can seek to recruit staff from other hospitals, less specialized in ID hospitals or redeploy nurses within the same hospital to supplement a Pyramid load. But this came with problems, given that it took some time to settle in staff into a new environment and/or role and for them to become efficient. Thus, (S3) drilling all existing staff in Infection Control policies before bringing in extra support was seen as important; this included setting up leadership roles and accountable training leads to coach new recruits effectively was one way to improve capability.

Similarly, thinking ahead about recruitment during peacetime was important. For example, it was suggested that (S4) CVs of short-listed clinical, nursing and lab applicants should be kept after recruitment drives, with permission, so that these candidates can be called on during surge events. There was discussion of using volunteers: ‘*We did have a meeting and many people volunteered, PhD people volunteered…but we decided not to go with that and just employ our own people and use our own people.’ - M1.* But the consensus was that the people with the right CVs for the post were needed, and it was better to build up a contacts list for these, or redeploy within, than stretch people’s capability.

The other facet of staffing is related to the social and psychological support strategies motivating *willingness to work*, and to work well, of healthcare professionals during these turbulent times. Related strategies are particularly important when facing virulent and deadly pathogens such as SARS or Ebola. Good leadership, (S5) motivating by example, was perceived to the most important factor keeping staff turning up and doing their best. During SARS the leaders volunteered to do the risky procedures and praised their staff to the media [15]. (S6) Transparent and honest feedback systems were seen to improve both work attendance rates and workflows and were described as one of the best ways of retaining the confidence from staff; this will be further elaborated communication section below.

(S7) Staff greatly valued the provision of adequate Personal Protective Equipment (PPE) and clear, repeat training and routines to protect themselves. During SARS for instance temperature of all working staff were taken at regular intervals. Ebola drills were deemed reassuring. Besides these practical considerations, a major theme in our participants’ narratives revolved around the cultural idea of hospital ‘kampong’ or village society, a notion retained from an earlier period in Singapore culture. This was described as a feeling of belongingness, ‘we are in this together’ spirit that helped in combating the fears of the staff. As one participant explained: ‘*you have a common goal, you have a common enemy, and between nurses, doctors, allied health admin… So many of us reflect this and find that it was a time rekindle our kampong spirit.’- BM2*. Offering staff morale boosting food and in some hospitals quarantine housing nearby was seen to go a someway to help (S8) maintain this caring and close-knit culture.

Still, the biggest demoralizing factor for staff were described as fears of infecting their families; particularly their children. Relatedly, one of the strategies for optimal infection control policy during SARS was to maintain what became referred to as ‘dirty – clean’ teams, the ‘dirty’ ones being the ones serving the infected patients and clean was serving non-SARS patients. Feeling of ostracism and not wanting to come in contact with family anyway caused a lot of additional stress: *‘We actually stayed at home—is that the best? What about my family?’- C3.* Such overt segregation can also lead to higher infection rates, due to lax PPM of the ‘clean’ teams.

(S9) Minimizing ostracism and streamlining practices of ‘dirty’ vs. ‘clean’ teams emerged as essential. During a comparatively empty hospital anyhow we propose that it is feasible for *all* staff to be encouraged to uptake routinized basic PPM, thus emphasising that anyone may have ‘dirty’ exposure. More stringent measures should, of course, be used for those workers in close contact with infected patients, as well as comfortable longer-stay accommodation giving colleagues the option to be matched with someone they know. Deliberate rewording of ‘close contact’ teams and ‘less exposed’ teams should be emphatically used.

#### Space

*Space,* a classic capacity issue, was unanimously characterised by reference to a system that was built to cater to chronic diseases, and nowadays emphasising solutions for an ageing population, in which ID has not been a priority. It is very difficult to create ID space in already congested hospitals: ‘*I think because [in] Singapore, you know, infectious diseases is not our number one disease burden, so a lot of way our infrastructure is structured might not actually cater for that [ID outbreak].’ - OM3.* Space has always been an issue in ID surge events where isolation measures pose an additional burden on the existing space crunch. Currently in Singapore there are few isolation rooms in the hospitals built before SARS and hence patients already exposed to each other (family) were cohorted in the same isolation room by partition. It was explained that it is part of the *Institutional role* to back the initiatives that (S10) counter this outdated historical legacy.

One solution has been to plan a new ID Centre, the National Centre for Infectious Disease (NCID), planned to be in operation by 2018 [22]. This building will be able to admit between 200 and 300 patients during an ID surge event. It has been suggested that paradoxically one ‘*cannot plan…you cannot build a white elephant’*, just on the off chance of it being needed. It has also been acknowledged that maybe it is not really needed, since alongside an ID surge there is a reduction in electives that free up bed capacity, also: *‘Of course if it is a ID surge, the general public will avoid us. So, then there will possibly be more room to reallocate our spaces and all that’ - C5*. Nevertheless, (S11) facing the ‘white elephant’ appeared to be underway, and staggered to make it manageable.

For example, senior administrators and clinicians discussed lobbying for more single occupancy isolation rooms, Microbiologists for enough space for their large equipment to process screening, and state-of-the-art equipment. In the meantime, the system has adapted by using temporary structures, such as setting up laboratories in general Intensive Care Unit (ICU) rooms (Ebola preparations). Alternate Treatment Sites (ATS) were built by hospitals as an emergency tented areas during H1N1. Such spaces are necessary to planning in a space constrained setting. *National policy* level initiatives to (S12) improve these set-ups include scouting better locations, as well as structures with capacity for greater distancing of patients, temporary ventilation systems and basic amenities.

Another solution to minimize congestion is to (S13) restructure insurance plans and primary care capacity to address overuse of hospitals for ID, instead moving screening and basic treatments to primary care clinics. In the long run this will create more hospital capacity without having to build it. It is a phenomenon particular to Singapore Health insurance Medisave policy [23], that it encourages the over-utilisation of hospitals because hospital bills are paid by insurance, whereas GPs and polyclinics visits are largely paid out of pocket as such: ‘*Because if you get admitted, you goes under Medisave, you know so if you get a full blood count every day, cost 30 dollars a day, and you know you get your….whatever, 40 bucks a day, times five days you know pay by Medisave. But if you went to a GP, charged you 40 bucks a day, you will pay out of your pocket’ - PH01.*

Another national level policy concern relates to much toing and froing on whether to have a designated hospital to house all patients during a nationwide outbreak or whether each hospital ‘keeps’ its own patients without referring them onwards. This question is not easy to address. During SARS one hospital - Tan Tock Seng Hospital (TTSH) - was designated. From a capability perspective, it appears more efficient to centre the bulk of resources in one place; yet we were reminded by our participants that this comes with risks (contagion during transportation and transfer), and requires planning of transport and investment in logistics. It is nevertheless underway (S14) that a state-of-the-art ID hospital is being built. Largely, it appears, to be able to deal with a Reverse-Pyramid hospital surge scenario. This new facility should particularly cater adequate isolation wards, unlike wards during SARS: ‘*So from a ward that is 30-35 bedder, suddenly you are dealing with ward with seven beds. Because there were only seven cubicles [and only one bed can be occupied in it at a time] and same number of staff.’- BM2*.

It was notable however that: ‘Making an acute hospital another pandemic hospital was not a viable idea... because as you know by now most of Singapore’s hospitals function at close to maximum capacity… And shutting down a hospital has tremendous inextricable impacts on the healthcare system’ - C4. A huge Pyramid or Black scenario surge would require reserve (S15) ‘sister’ hospitals and centres to manage overspill. Some participants mentioned that collaboration with private hospitals could be helpful, and something that was not yet leveraged. Under-utilized primary care centres and smaller public sister tertiary centres can be set up to function as a centralised hub during a mass outbreak. During H1N1 overspills were naturally managed with sister-like facilities, adopted opportunistically as the need arose.

It was felt that the selection of appropriately regionally distributed centres and coordination of planning and response during Peacetime would minimise disruption to on-going services during an outbreak. Nevertheless, there are known drawbacks with using a designated ID hospital, not least its likelihood to become stigmatised for use during peacetime, the potential shutdown of multiple large hospitals was not viable. It was however notable to us that the biggest drain on the system appeared to be the lack of confirmed decision on this policy - which resulted in stockpiling and planning for a ‘what if we have to cope with this alone’ scenarios, which can be seen as siloed, duplicative and ultimately wasteful strategies.

#### Supplies

As for *supplies,* we were told about improvements in ID surge preparedness *Equipment and stockpiling* since SARS, during which a shortage of everyday supplies for patient management (X-ray films, PPEs, etc.) was felt. When H1N1 pandemic hit, the access to resources were described as ready and available. Current policy in many hospitals was to (S16) independently stock PPE’s, drugs for at least 3 days, in order to allow time to reorder in case of a surge event. In addition, (S17) transparent listing of reserve supplies on a centralized system is important for coordinated planning. For example, to know availability of surplus medical supplies and planning for ID outbreak equipment will help operations managers assess shortfalls, particularly if they are designated a sister centre during a mass Pyramid or potentially Black outbreak. (S18) Operations managers spoke of valuing good relations with international suppliers.

Certain hospitals have also reported *Financing for innovation*, in particular (S19) investing in automated devices to facilitate the monitoring of patients for clinical management: *‘In terms of equipment we know that it will be much better with using more automated devices, instead of manually measuring’*- BM2. Regarding systems investments, paradoxically, OMs tended to feel their budgets were realistic, even generous: *‘I am glad to say, and this can go on record, that MoH is very generous with the budget and especially when it comes to an outbreak.’ – OM1.* Whereas medics expressed reservations along the lines (S20) that there was too much emphasis on frugality, or basic care delivery, above innovation and effectiveness: *‘The tagline is Singapore healthcare, the most cost effective. [but] The emphasis is on* ***cost*** *[emphasis their own] effective. Not effectiveness. So it is bang for buck’ - M3.* Placing effectiveness above short term cost-saving would improve capability in the face of any surge threat.

#### Systems

*S* ***ystems*** were built on generally sound communication, emphatic consideration of logistics and operations and some efforts toward monitoring and surveillance. With respect to *Communication* it was relayed that Singapore higher management (S21) valued principles of transparency. During SARS *‘The management was actually brutally honest’- C1 [on SARS]*. One poignant example was shared during H1N1, when the only available Tamiflu was recorded as past ‘best date’. Rather than try and cover this up the management checked the efficacy of the drug and was upfront about the issue: ‘*basically we went on TV say you know this stuff is perfectly good, we’ve seen the test, someone saying ‘I would take it and give it to my kids’.’PH1*. This open dialogue with the public built trust as well as transparency into the system.

In contrast, within institutional structure the flow of communication has been very top-down with members assigned an authoritative person to take instructions from. It was noted that the system might benefit from (S22) a flatter and wider structure. It was shared that with fast moving issues on the ground, top-down instructions may not be applicable; some system to feed how to adapt upwards was required. Immediate notification was issued to senior staff, especially to doctors via email. Nurses didn’t have any institutional email were communicated only by roll calls at the start of each of the three shifts in a day. One way to improve this was suggested via SMS notification that could send alert and procedural notifications direct to mobile phones.

A response inbox could be set up for staff to upload notifications of difficulties on the ground, addressing the general malaise that the decision makers: *‘[MoH] are in an ivory tower… the layer between them is the hospital administrators, of course…’-C6*. As for boarding of communication channels - it was noted that professionals were quick to come together in a crisis - but it was emphasized that inter-agency communication needs to also happen in Peacetime, and share information. Relatedly, *Monitoring and surveillance* would also be helped by (S23) recording bottom up bottlenecks, preferably in real time. Surveillance in Singapore as an island nation has otherwise been actioned toward uniformity, through liaison with ministries. During major outbreaks Singapore is notable as one of the few countries to issue daily surveillance report against the standard one-week reports in other pandemic affected countries.

**Fig. 4:**
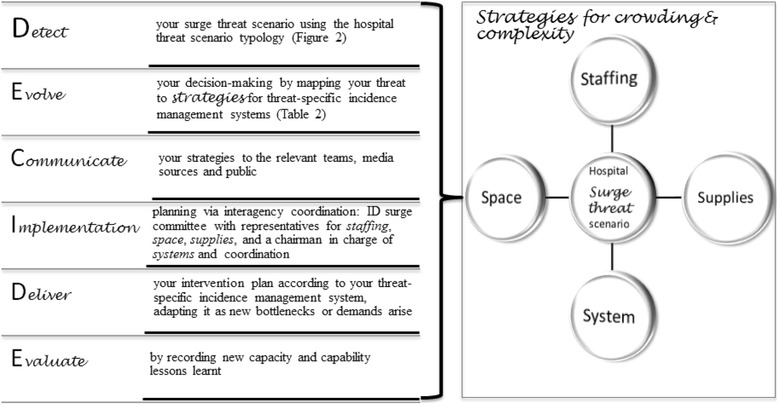
DECIDE steps to coordinate planning and response for hospital ID surges

Yet (S24) apart from prevalence data, other more meaningful datasets and collaborative efforts to inform future outbreak modelling appeared scarce and reluctant to surface. Participants also mentioned the hierarchical nature of system as a deterrent in vocalizing their thoughts and working together in research areas. There were also (S25) concerns regarding the detection of an unusual cases or outbreak as there has been no formal system set up to do this, or generalizable tools to guide what to do in response; each outbreak was seen to require its own disease-specific protocol, intuitively and implicitly using lessons learnt – this highlights the need for the DECIDE tool proposed herein (see discussion section and Fig. 4). We were told that as a rule of thumb an incident of more than five cases of a particular disease, made it notifiable, but this not a formal guideline. Although much emphasis was based on notifiability, the real issue with using these data during an ID outbreak was to help detect the threat, predict and plan logistics.

*Logistics & operations* were underscored as such: ‘*In terms of [the surges] stress on the system, [it impacts] not in a emotional, or clinical, or structural [sense], or even…in terms of science, as much as on the logistics’ - BM3*. But logistics for what? Without a reference point and with changing guidelines logistics and their operations are simply dress rehearsals for ‘what if’ scenarios that never to materialise. Here it became apparent that mapping the threats and how people can share resources to address them was central to optimising logistics. Every hospital has an emergency-planning department that stays alert and issues protocol during surges, which is then managed across departments to make HCWs respond in a coordinated manner. These systems need to be harnessed as efficiently as possible. Infection Control (IC) and to a lesser extent complex disease management were at the centre of these systems.

Such planning tended to boil down to capacity or (S26) ‘upsizing’ to address IC by increasing staff ratios because of lengthy, time consuming, strict infection control practices; or to deal with travellers brought for screening and isolation at the hospitals straight from airport; added paperwork, liaison with the embassies. But there were also the months of capability assurance and training. Learning about equipment and treatment protocols, running PPE usage drills, practicing patient transport routes. Transferring of suspected and probable cases across hospitals remains a major area of concern. Contact tracing of taxi drivers and passengers once SARS was known may well have saved Singapore from a Black scenario. Standardizing these processes, including mortuary procedures and sewage cleaning, and limiting a bulk of resources to designated and sister hospitals can save procedural waste.

#### Decision-making

Regards decision-making we noted that leaders of Singapore are very principle driven; and expect policies to be based on fundamentals of science; but science in context, relying on institutional memories, as one operations manager put it: *‘You know in Singapore, we work things out from first principles, we will already have some certain level of knowledge as to what will work and what will not work.’- OM2*. The efficacy of ministries was respected. Targeted involvement from health professionals in the formulation of policies during surges was also seen to be an important exercise. The worry was also the institutional memories were getting lost: ‘*And I think immediately after SARS, the awareness was there for everyone but there been turnover and everything. So, people have actually, you know, kind of like forgotten you know, so that is one thing’ - OM3.*

As such we propose to formalise institutional memories, using them systematically toward better decision-making. Systems function rests ultimately on good coordination, which is not an exact science, takes account of logistical realities while using institutional memory. This is but likened to an engineering challenge: *‘I am putting it to you that a surge response is an engineering challenge. It is not a science challenge. It is not an admin challenge. It is not a microbiology or lab challenge. It is an engineering challenge.’ –M2.* Like all engineering challenges the apt response depends on the soundness and extent of understanding of the problem, finding a mapped solution, communicating and sharing it accurately and adapting it when needed*.*

## Discussion

### Using DECIDE steps

To preserve, advance institutional memories, and ensure new ones are captured we propose the DECIDE steps. The six steps in DECIDE, are detailed in Fig. 4, and are summarised as: **D**etecting, and linking to surge threat scenarios, as defined in Fig. 2; **E**volving, by linking your threat to strategies, Fig. 3; **C**ommunicating your approach to your various stakeholders; **I**mplementation planning through an appropriately representative committee (in terms of staff, space, supplies and systems); Delivering your intervention by adapting it to unexpected bottlenecks; and again – since nothing ever fully goes to plan - **E**valuating it, by recording new lessons learnt and updating your list of strategies as you go.

Some institutional memories will be instantly clear and can be brought to immediate systems benefit. Others, may require time - professional, disciplinary and cultural distancing to come into focus. It is these memories and experiences, which this research sought to record. One such notable conclusion is that the existing constraints on the Singapore hospital systems, operating at maximum capacity itself appears to have rendered it buoyant to shocks, rather than more vulnerable to them. People, departments, agencies have had to learn to adapt every day to never lessening demands. This itself seems to have rendered the system better able to absorb and bounce back from shocks. Shocks such as H1N1 could have utterly collapsed a health system with existing space crunches.

### Main findings

It was a surprising finding that Dengue or other endemic and seasonal surges were absorbed by a system so close to the brink, in such a normalised fashion. It is nevertheless both paradoxical yet somehow evident that a system which is so clearly burdened at the outset in terms of capability is able to respond so well to it in a time of crisis. Capacity is a systems’ issue that Singapore is conquering; perhaps issues such as space and manpower influxes are simply easier to solve in the interim than capability related ones. These, in turn, are evidenced to be enabled by flatter, more explicit structures and leveraging of more integrated services and ways of working.

Conceptualization of ID hospital surges will help toward better, practical, pre-emption of problem areas [14, 15]. It has been demonstrated that Past ID surge events responses were compromised due to lack of coordination between hospitals leading to underutilization of resources [5–9, 14, 15]. Singapore has taken considerable steps towards creating adequate preparedness for an ID outbreaks and related hospital surges, like hiring additional ID staff and training them in infection control activities [16]. It has also planned having a buffer in additional staff [24] and redeploying staff trained in infection control in the same institution, which has been demonstrated to be better than relying on volunteers in earlier studies [8, 14]. Staff using their tacit knowledge, working flexibly and responsibly rather than being overstretched; plus, sticking to incident management protocols has been shown to be optimal in management of ID surge events [25].

Hospitals and social institutes serve as amplifiers of ID spread [13]; during SARS and MERS incidents of spreading infections to healthcare workers were at their highest. Previous studies show that provision of adequate PPEs, infection control training helped the staff feel confident enough to stay put [26, 27] in any ID outbreak despite the fact that they knew the high risk of contracting infection. These measures combined with transparent communication and showing genuine concern for the welfare of their staff helped in building trust and confidence in their institution [26–29]. Still, the majority fear infecting their family, and themselves, and being ostracized. Segregation of ‘dirty’ vs. ‘clean’ teams has been shown to fail in terms of IC, where the teams least exposed were ultimately most likely to get infected [30].

Complete shutdown of mainstream hospitals, as was the case during SARS in our setting and others, has shown difficulty in recovering [31] and to remain stigmatized by ID associations. Transportation of suspected cases from one hospital to another is another big area of concern. Ambulatory personnel can be infected while transferring patients from one hospital to other [30]. There were divided opinions on the usefulness of a single centre taking charge of ID surge. Yet to have a designated ID hospital equipped with the right capability and improving the capability of chosen ‘sister’ institutions will save total shutdowns and duplications of planning measures. Singapore has planned a centre solely for ID [22], this coupled with creation of appropriately selected and provisioned alternate treatment sites, and cutting down of electives [32] keeps the system working a as whole. Peripheral hospitals tend to have fewer ID staff, nor funding to establish high capability in handling IDs [4, 5, 33, 34].

Developing a robust technology in preventing infection control mishaps and e-health helping in detecting patterns will help deal better with nosocomial infections and intervening early [35, 36] This approach was recently highlighted by the Minister of State for Health Chee Hong Tat who announced plans to make better use of IT systems and data analytics to help them make better decision [37]. And ways of using these in a shared and transparent manner needs to be agreed. The need for better inter-agency coordination, using institutional memory and in particular closing the gap between administrators and clinical staff voices, is a common finding across setting [5, 6, 38]. The said ‘over-reaction’ to H1N1 – mass screening policy – which clinical staff noted to be based on an overly cautionary reading of the diseases threat is one example of this.

#### Strengths and limitations

Our Framework for ID hospital surge planning & response builds on known elements and supplements this existing work with primary, targeted, qualitative analysis. Although many evaluations of surge management will have documented disparate strategies, in specific contexts, to our knowledge the conceptually mapping, making use of historical data and institutional memory has not yet been undertaken.

While all the 26 strategies that we identified as useful in the Singapore context may not be generalizable to all countries, the conceptual elements grouping them and identified typology threat scenarios are adaptable to different settings. We contend that the steps in the DECIDE model are transferable and offer a useful process to map hospital ID surge planning and response in any context.

We were limited by recruitment largely in one main hospital (TTSH), because this is the hub of ID specialism in Singapore, although remaining structures in Singapore comprised nearly half of our interviewees. Singapore General Hospital (SGH) refused to participate.

## Conclusions

In sum, the current analyses are intended to help us learn about the specific to inform the general. Our Peacetime scenarios (inclusive of manageable endemic outbreaks), Pyramid, Reverse Pyramid, and Black scenarios will be catalysed by different IDs in different settings. For example, in settings that cannot prevent vector outbreaks at source so well, endemic outbreaks - e.g. Malaria during the rainy seasons - may not be manageable, and will instead be typified as a pyramid scenario. Likely with more admissions than was the case with H1N1 in our setting due to higher transmissibility, thus a wider middle tier and admissions peak. And not all Peacetime settings will be predisposed to capacity limitations from the start. The current study fills in the shapes of what is ‘known’ to help plan for ‘unknowns’ [39] during ID outbreaks.

## Abbreviations

ASEAN: Association of South East Asian Nations
BM: Board members
C: Clinicians
CGH: Changi General Hospital
COREQ: Consolidated Criteria for Reporting Qualitative research
CV: Curriculum Vitae
ED: Emergency Department
GP: General Practitioner
ID: Infectious Diseases
IntD: Interpretive Description
KTPH: Khoo Teck Puat Hospital
M: Microbiologist
MERS CoV: Middle East Respiratory Syndrome Coronavirus
MoH: Ministry of Health
N: Nurses
NUH: National University Hospital
OM: Operations Manager
PHP: Public Health Practitioners
PPE: Protective Personal Equipment
PPM: Protective Personal Management
SARS: Severe Acute Respiratory Syndrome
SGH: Singapore General Hospital
TTSH: Tan Tock Seng Hospital
